# Expression Pattern and Clinicopathological Relevance of the Indoleamine 2,3-Dioxygenase 1/Tryptophan 2,3-Dioxygenase Protein in Colorectal Cancer

**DOI:** 10.1155/2016/8169724

**Published:** 2016-08-08

**Authors:** I-Chien Chen, Kuen-Haur Lee, Ying-Hua Hsu, Wei-Ran Wang, Chuan-Mu Chen, Ya-Wen Cheng

**Affiliations:** ^1^Department of Life Sciences and Agricultural Biotechnology Center, National Chung Hsing University, Taichung 402, Taiwan; ^2^Graduate Institute of Cancer Biology and Drug Discovery, College of Medical Science and Technology, Taipei Medical University, Taipei 110, Taiwan; ^3^Cancer Center, Taipei Medical University Hospital, Taipei Medical University, Taipei 110, Taiwan; ^4^Department of Chemical Engineering, National Tsing Hua University, Hsinchu 300, Taiwan; ^5^Graduate Institute of Medical Sciences, Collage of Medicine, Taipei Medical University, Taipei 110, Taiwan

## Abstract

*Aims*. Cancer cells use the indoleamine 2,3-dioxygenase 1 (IDO1) pathway to suppress the host's immune response in order to facilitate survival, growth, invasion, and metastasis of malignant cells. Higher IDO1 expression was shown to be involved in colorectal cancer (CRC) progression and to be correlated with impaired clinical outcome. However, the potential correlation between the expression of IDO1 in a CRC population with a low mutation rate of the* APC* gene remains unknown.* Material and Methods*. Tissues and blood samples were collected from 192 CRC patients. The expressions of IDO1, tryptophan 2,3-dioxygenase (TDO2), and beta-catenin proteins were analyzed by immunohistochemistry. Microsatellite instability (MSI) was determined by PCR amplification of microsatellite loci.* Results*. The results showed that high IDO1 or TDO2 protein expression was associated with characteristics of more aggressive phenotypes of CRC. For the first time, they also revealed a positive correlation between the abnormal expression of beta-catenin and IDO1 or TDO2 proteins in a CRC population with a low mutation rate of* APC*.* Conclusion*. We concluded that an IDO1-regulated molecular pathway led to abnormal expression of beta-catenin in the nucleus/cytoplasm of CRC patients with low mutation rate of* APC*, making IDO1 an interesting target for immunotherapy in CRC.

## 1. Introduction

Colorectal cancer (CRC) is a leading cause of cancer-related deaths worldwide. There are nearly one million cases of CRC diagnosis worldwide each year [1, 2]. CRC development is a complex and multistage process, resulting from genetic mutations and environmental factors [3, 4]. The genetic influences in CRC progression occur at various points and include* APC* mutations in the early stages and mutations in tumor suppressor* TP53* and oncogene* KRAS* during the later stages [5]. These mutations are thought to drive malignant epithelial transformation. Mutations inactivating the* APC* tumor suppressor gene are believed to be critical in the majority of colon adenomas and carcinomas [6]. The incidence of* APC* mutations varies widely among populations. Approximately 70%–80% of sporadic colorectal adenomas and carcinomas have somatic mutations that inactivate APC in Western countries [7]. In colorectal adenomas and carcinomas where both* APC* alleles are defective, destruction of the free pool of beta-catenin is impaired, and active beta-catenin accumulates in the cytoplasm and nucleus to reactivate Wnt/beta-catenin target genes in CRC [8]. Our previous study showed that mutation rate of the* APC* gene in CRC was 33.8% [9], which is close to the reported level in Asia [10, 11] but significantly lower than in Western countries [7]. Collectively, these results suggest that another mechanism exists for the regulation of CRC carcinogenesis.

Immune evasion is a hallmark of cancer and involves complex mechanisms, which enable cancer cells to evade the host's immune system [12]. Cancer cells use indoleamine 2,3-dioxygenase 1 (IDO1) pathway to suppress the host's immune response in order to facilitate survival, growth, invasion, and metastasis of malignant cells [13]. IDO1 can catalyze the oxidative catabolism of tryptophan (Trp) to kynurenine (Kyn) [14]. IDO1 exhibits its immunosuppression effect by suppressing the response of macrophages and effector T cells via Trp starvation of sensitive T cells or the build-up of toxic metabolites (Kyns) from Trp metabolism, leading to cell cycle arrest and death of effector T cells within the cancer cell microenvironment [15]. IDO1 activity also directly promotes tumor growth and proliferation of the neoplastic epithelium in a cell-autonomous fashion via the generation of Kyn metabolites and activation of beta-catenin signaling [16]. Previous study revealed that higher IDO1 expression was involved in CRC progression and correlated with impaired clinical outcome [17]. However, the correlation of IDO1 expression in population with a low mutation rate of the* APC* gene remains unknown.

The present study aimed to investigate a possible prognostic role of IDO1 and Kyn and Trp metabolites in clinical CRC tumors and analyze the correlations between expression level IDO1 and mutation status of* APC*,* TP53*, and* KRAS* genes of CRC tissues. The correlation of the distribution of the beta-catenin protein or microsatellite instability (MSI) with IDO1 expression in clinical CRC tumors was also investigated in this study.

## 2. Materials and Methods

### 2.1. Patients and Specimens

CRC tumor tissues were collected from 192 nonselected patients who underwent surgical resection for CRC in the Department of Surgery, Taipei Medical University Hospital, between August 2012 and 2014. Informed written consent was obtained from all patients and/or guardians to use of their resected specimens. The acquisition of samples and their subsequent examination were approved by the Institutional Review Board of Taipei Medical University. None of the participants had a previous history of cancer. The clinical stages and pathological features of primary tumors were defined according to the criteria of the American Joint Committee on Cancer.

### 2.2. Immunohistochemical (IHC) Analysis

Paraffin-embedded sections (3 *μ*m-thick) of the tissue sections were prepared and stained with H&E for histological analysis. All the tissue sections were stained with a standard IHC protocol. Briefly, slides were deparaffinized by serial xylene-ethanol treatment. Antigens were retrieved by boiling in sodium citrate buffer for 10 min. Slides were blocked in 5% normal goat serum for 1 h at room temperature. After blocking, the slides were incubated with a primary antibody against IDO1, TDO2, and beta-catenin, followed by a biotin-conjugated secondary antibody, polymer-horseradish peroxidase, and a diaminobenzidine tetrahydroxychloride solution. The intensity of staining was scored as follows: 0 point, negative; 1 point, weakly positive; 2 points, moderately positive; or 3 points, strongly positive. The percentage of positive tumor cells (0–100%) was multiplied by the intensity of all proteins staining; therefore, the overall score ranged from 0 to 300. The proteins expression scores (0–300) were divided into two groups: low expression (0–150) and high expression (151–300).

### 2.3. Measurements of Serum Levels of Trp and Kyn Metabolites in CRC Patients

The majority of blood samples were collected locally at the Taipei Medical University Hospital. Blood samples were collected from 192 patients in serum tubes with gel separator. Free Trp and Kyn serum concentrations (*μ*M/nM) were determined by high-performance liquid chromatography (HPLC), as described [18].

### 2.4. *APC*,* TP53*, and* KRAS* Genes Mutation Analysis

Genomic DNA was prepared from 172 OCT-embedded frozen CRC tissues using standard proteinase K digestion and phenol/chloroform extraction after homogenization. Mutations in* APC*,* TP53*, and* KRAS* genes were determined by direct sequencing of polymerase chain reaction (PCR) products. Target sequences were amplified in a 50 *μ*L reaction mixture containing 20 pmol of each primer, 2.5 units of Taq polymerase (Takara Shuzo, Shiga, Japan), 0.5 mmol/L dNTPs, 5 *μ*L PCR reaction buffer, and 1 *μ*L genomic DNA as the template. Four sets of oligonucleotide primers for* APC* (forward: 5′-CAGACTTATTGTGTAGAAGA-3′ and reverse: 5′-CTCCTGAAGAAAATTCAACA-3′; forward: 5′-AGGGTTCTAGTTTATCTTCA-3′ and reverse: 5′-TCTGCTTGGTGGCATGGTTT-3′; forward: 5′-GGCATTATAA-GCCCCAGTGA-3′ and reverse: 5′-AAATGGCTCATCGAGGCTCA-3′; forward: 5′-ACTCCAGATGGATTTTCTTG-3′ and reverse: 5′-GGCTGGCTTTTTTGCTTT-AC-3′); three sets of oligonucleotide primers for* TP53* (forward: 5′-TGCCCTGAC-TTTCAACTCTG-3′ and reverse: 5′-AGTTGCAAACCAGACCTCAGG-3′; forward: 5′-CCTGTGTTATCTCCTAGGTTG-3′ and reverse: 5′-TCTCCTCCACCGCTTCT-TGT-3′; forward: 5′-AAGGCGCACTGGCCTCATCTT-3′ and reverse: 5′-GAATCT-GAGGCATAACTGCAC-3′); and one set of oligonucleotide primers for* KRAS* (forward: 5′-AGGCCTGCTGAAAATGACTGAA-3′ and reverse: 5′-AAAGAATG-GTCCTGCACCAG-3′) were used to amplify the mutation cluster region of* APC*,* TP53*, and* KRAS* genes, respectively.

### 2.5. Microsatellite Instability (MSI) Analysis

MSI was determined by PCR amplification of microsatellite loci [19] from DNA extracted from tumors. A high degree of MSI (MSI-high) was defined as the presence of instability in 30% of the markers, and MSI in <30% of the markers were defined as MSI-Low.

### 2.6. Statistical Analysis

A chi-square test was used to compare IDO1 and TDO2 protein expression with clinicopathological parameters, correlation between the expression of IDO1 and TDO2, correlation between mutations of genes and IDO1 or TDO2 protein expression, correlation between IDO1 or TDO2 protein expression and beta-catenin protein distribution, and correlation between IDO1 or TDO2 protein expression and MSI. Kaplan-Meier survival curves were constructed for overall survival (OS) and progression-free survival (PFS) to evaluate the survival curve difference between low or high expression levels of IDO1 or TDO2 proteins. A probability of <0.05 was considered statistically significant. All statistical analyses were performed using SPSS software (SPSS, Chicago, IL, USA).

## 3. Results

### 3.1. The Expression of IDO1 and TDO2 Was Significantly Increased in CRC Tissues and Correlated with Lymph Node Metastasis and Tumor Stage of CRC

Both IDO1 and TDO2 triggered by an immune challenge can catalyze Trp to Kyn and then start Kyn pathway [20]. To understand the clinical relevance of IDO1 and TDO2 in CRC, IHC analysis was performed to analyze the protein expression of IDO1 and TDO2 in 192 human CRC tissues. The distributions of demographic, clinical, and pathologic features are presented in Table 1. The results showed that the expression level of IDO1 (Table 1) was associated with gender (*p* = 0.042), node metastases (*p* = 0.047), and clinical staging (*p* = 0.027). However, no statistically significant relationship was found between IDO1 expression and age, tumor depth, or metastasis. In addition, a significant association between the expression level of TDO2 and tumor depth (*p* = 0.039), node metastases (*p* = 0.028), and clinical staging (*p* = 0.012) was found in 192 human CRC tissues (Table 1). However, no statistically significant relationship was found between TDO2 expression and age, gender, and cancer staging. Further, the correlation between IDO1 and TDO2 expression in the tumor tissues of the CRC patients was analyzed. As shown in Table 2, the expression of IDO1 was significantly positively correlated with the levels of TDO2 (*p* < 0.0001). Collectively, these data indicated that IDO1 and TDO2 could serve as prognostic markers in CRC and that they were strongly correlated with protein expression levels and clinical features of advanced disease.

### 3.2. IDO1 Activity Was Determined in the Serum of CRC Patients

Next, to understand the activity of IDO1 in the CRC population, IDO1 activity was estimated by detection the levels of Kyn and Trp in serum. To evaluate IDO1 activity of CRC patients, the concentrations of Kyn and Trp were measured by HPLC in each individual.  Table 3 shows the results of the analysis of the relationship between Kyn or Trp plasma concentrations and clinicopathological parameters, such as age, gender, histological grade, stage of the disease, and the presence/absence of metastasis. No significant relationship was found between the plasma Kyn or Trp levels and any of the clinicopathological parameters, except MSI.

### 3.3. Correlation of IDO1 and TDO2 Expression and Genes Mutations in CRC Patients

Studies of tumor tissues with genetic alterations in* APC*,* TP53*, or* KRAS *alone could help identify the way in which IDO1 regulates these genetic alterations [21–23]. To understand whether alterations in* APC*,* TP53*, or* KRAS* genes were correlated with IDO1 or TDO2 expression, tumor tissues were collected from 172 CRC tissues. Of the genes studied,* APC *mutations were found in approximately 42% of the samples studied (Table 4), in agreement with our previous report [9].* TP53 *and* KRAS *genes were the least frequently mutated of the genes studied, with approximately 20% of tumors affected (Table 4). Moreover, no significant association was found between* APC*,* TP53*, or* KRAS *gene mutations and IDO1 or TDO2 expression (Table 4). These data demonstrated that genetic alterations in* APC*,* TP53*, or* KRAS *were not associated with the expression status of IDO1 or TDO2 in CRC.

### 3.4. Correlation of the Expression of IDO1 and TDO2 and the Distribution of the Beta-Catenin Protein

Previous studies demonstrated that a high accumulation rate of active beta-catenin in the cytoplasm and nucleus due to the* APC *mutation was linked to the initiation of colorectal tumorigenesis in Western countries [24, 25]. To understand the accumulation rate of beta-catenin in the cytoplasm and nucleus of low* APC* mutation population in Taiwan, the expression and distribution of beta-catenin protein in 192 CRC tumors were examined by IHC analysis. As shown in Table 5, up to 54.7% of beta-catenin was detected in the cytoplasm and nucleus. This observation suggests that the* APC* mutation is not fully responsible for active beta-catenin in the cytoplasm and nucleus and consequently for the CRC tumorigenesis. Recently, beta-catenin activation by IDO1 has been demonstrated in an animal model of colitis-associated CRC [16]. However, this phenomenon has not yet been examined in clinical CRC patients. Thus, the association between the expression of IDO1 and TDO2 and beta-catenin was investigated in clinical CRC patients. As shown in Table 5, the expression of IDO1 or TDO2 was significantly positively correlated with active beta-catenin in the cytoplasm and nucleus (*p* = 0.020 for IDO1; *p* = 0.043 for TDO2). These results suggested that IDO1 was involved in the activation of beta-catenin in a low* APC* mutation population.

### 3.5. Relationship between IDO1 or TDO2 Expression and MSI

MSI refers to altered lengths of short nucleotide repeat sequences in tumor DNA compared with normal DNA [26]. Approximately 15–20% of CRCs are characterized by high-level MSI, and high-level MSI has been shown to be associated with abnormal protein expression [27–29]. Consistent with other studies, in the present study, high-level MSI was present in approximately 15% of CRC cases (Table 6). In addition, no significant association was found between IDO1 or TDO2 expression and MSI (Table 6).

### 3.6. Prognostic Relevance of IDO1 or TDO2 Expression Level for CRC Patients

**Figure 1:**
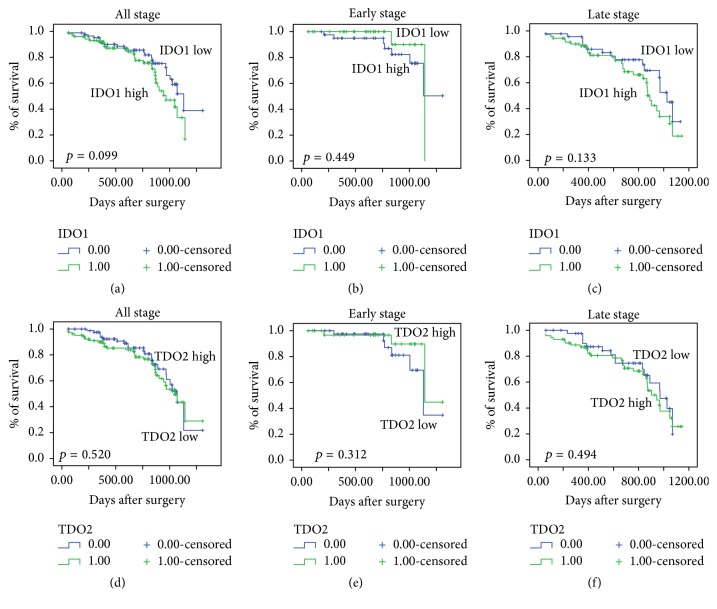
Correlation of IDO1 or TDO2 expression with the OS (overall survival) rate of CRC (colorectal cancer) patients. A Kaplan-Meier analysis of the OS among 192 CRC patients was performed according to the expression of IDO1 (a)–(c) and TDO2 (d)–(f) in tumor tissues of different disease stages.

**Figure 2:**
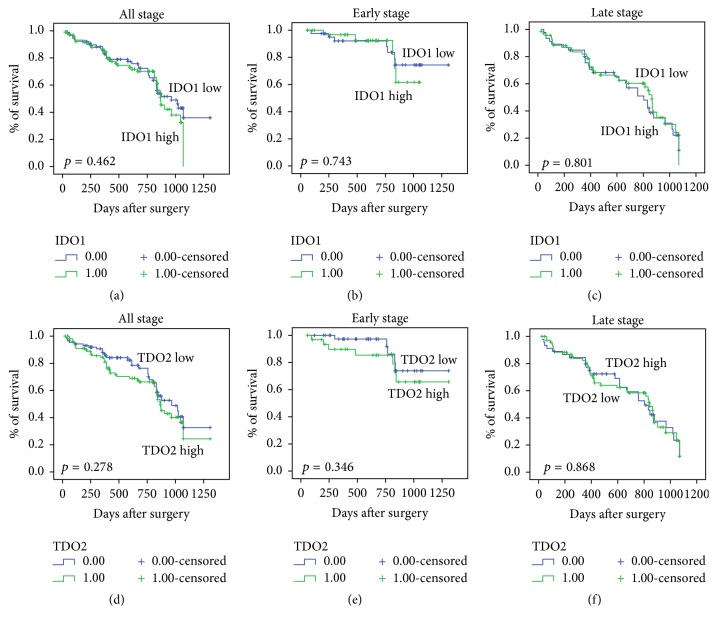
Correlation of IDO1 or TDO2 expression with the PFS rate of CRC patients. A Kaplan-Meier analysis of the PFS among 192 CRC patients was performed according to the expression of IDO1 (a)–(c) and TDO2 (d)–(f) in tumor tissues of different disease stages.

To clarify the correlation between IDO1 or TDO2 expression and overall survival (OS) or progression-free survival (PFS) of CRC patients, IDO1 or TDO2 protein expression was evaluated by IHC in 192 paraffin-embedded CRC tissues. According to the cut-off, low IDO1 or TDO2 expression was detected in 82/192 (45.8%) CRC tumors, and high IDO1 or TDO2 expression was detected in 104/192 (54.2%) CRC tumors. Analysis of Kaplan-Meier curves showed that high IDO1 or TDO2 expression was not associated with poor OS (Figure 1) or PFS (Figure 2) rates in patients with different stages of CRC.

## 4. Discussion

IDO1 and TDO2 play important roles in mediating both tumor immunoescape and immune response regulation [20]. Tumoral IDO1 or TDO2 expression was reported to be correlated with a poor prognosis in several types of tumors [20, 30, 31], which makes IDO1 and TDO2 as interesting targets for cancer immunotherapy. In this study, high IDO1 or TDO2 protein expression was associated with characteristic of more aggressive tumoral phenotypes. Moreover, we identified the positive correlation between abnormal expressions of beta-catenin and IDO1 or TDO2 proteins in the CRC population with low mutation rate of* APC*. Although a high mutation rate of* APC *is known to cause overexpression of beta-catenin in CRC patients in Western countries [7], it is unclear whether a low mutation rate of* APC *can cause overexpression of beta-catenin in CRC patients in an Asian population. The loss of* APC* alone is not sufficient to promote aberrant Wnt/beta-catenin signaling [32, 33]. Therefore, mechanisms other than* APC* mutation could be involved in activation of beta-catenin during colorectal tumorigenesis. A recent study demonstrated that IDO1 activity directly promotes colon tumorigenesis in mice model via the activation of beta-catenin signaling [16]. The present clinical study provides the first evidence that that overexpression of beta-catenin in a CRC population with a low mutation rate of* APC* takes place via IDO1-mediated regulation.

In the current study, the serum concentrations of Kyn and Trp were simultaneously measured using HPLC to determine the IDO1 activity in patients with CRC. The results revealed increased concentrations of Kyn in the group of CRC patients with clinical features of advanced disease, including late clinical staging, high tumor depth, and node metastases, but decreased concentrations of Trp in this group (Table 4). These data suggest that Trp catabolism by the IDO1 activity is associated with the progression of CRC. Different physiological and pathological factors, such as pregnancy, organ transplants, autoimmune diseases, or viral infections, are known to affect serum or plasma levels of Kyn in CRC patients [34, 35]. The aforementioned explains the lack of any significant association between Kyn or Trp concentrations and clinicopathological parameters in the present study.

A genetic model proposed by Fearon and Vogelstein for CRC tumorigenesis to describe the accumulation of genetic changes necessary to drive the transition from adenoma to carcinoma in the development of CRC has become generally accepted as a paradigm for the genetic basis of CRC development [6, 36]. The major genetic pathways of CRC are the inactivation/mutation of the tumor suppressor genes* APC* and* TP53* and activation/mutation of the oncogene* KRAS* that are required for tumor initiation and progression of CRC [37–39]. The present study analyzed these three key genes involved in CRC genesis. In this Taiwanese population, the frequency of* APC* mutations in tumor tissues was 42.4%. The incidence of* APC *gene mutations in Asian populations was previously reported to be 26–42% [9, 11, 40], whereas it was 37–56% in European populations [41, 42] and about 60% in US populations [43]. Thus, the mutation frequency of* APC *in the present study fell within the range reported in Asian populations but was lower than that in European or US populations, pointing to the potential role of an IDO1-regulated molecular pathway in tumor formation in Asian populations. The mutation rate of* TP53 *gene was reported to be about 34–53% in Asian populations [11, 44], 35–60% in European population [41, 45, 46], and 45.4% in US populations [47]. However, in the present study, the mutation rate of* TP53 *was quite low (just 18%). The low frequency of* TP53* mutation may be due to different dietary patterns, environmental conditions, and population differences. Finally, the mutation rate of* KRAS* gene was reported to be about 29–62.9% in Asian populations [11, 44], 25–38% in European populations [48–50], and 33–39% in US populations [51, 52]. The mutation rate of* KRAS *was 21.5% in the present study. Although this was lower than that reported in Asian, European, and US populations, it is similar to that reported in another study [53]. Collectively, the results suggest that environmental factors, such as diverse lifestyles and dietary habits, in addition to variable exposure to carcinogens, have a major impact on the mutation rate in different populations.

Accumulating evidence suggests that genetic aberrations induced by environmental, lifestyle factors, and dietary habits contribute to CRC tumorigenesis [54]. To establish the complex relationships among etiological factors, molecular alterations, and disease evolution, “epidemiology” and “molecular pathology” have recently become integrated, generating the interdisciplinary field of “molecular pathological epidemiology (MPE)” [55, 56]. MPE can address the question of how lifestyle or genetic factors interact with tumor molecular features to influence tumor cell behavior (prognosis or clinical outcome) in CRC [57]. In the present study, we revealed that high expression of IDO1 or TDO2 proteins was one of the causal factors to cause aberrant accumulation of beta-catenin in a CRC population with a low mutation rate of* APC*. Our study provides insights into MPE research about link between tumor molecular changes in CRC and hosts immune response and ultimately provides key concepts for application in patient-tailored therapy.

## 5. Conclusions

The present study found that increased expression of IDO1 and TDO2 was correlated with features of advanced clinical disease in CRC patients. Furthermore, the results indicated that an IDO1-regulated molecular pathway could possibly lead to abnormal expression of beta-catenin in the nucleus/cytoplasm of CRC patients with a low mutation rate of* APC*, suggesting that IDO1 is an interesting target for immunotherapy.

## Figures and Tables

**Table 1:** Association of IDO1 and TDO2 expression and clinical parameters in tumor tissues of CRC patients.

Parameters	IDO1		TDO2	
	Low	High	Low	High
	(*n* = 88)	(*n* = 104)	(*n* = 88)	(*n* = 104)
Age (years)				
≦65	42	52	42	52
>65	46	52	46	52
*p* value	0.774		0.774	
Gender				
Female	34	56	41	49
Male	54	48	47	55
*p* value	0.042^*∗*^		1.000	
T factor				
1	6	1	6	1
2	11	17	11	17
3	53	57	55	55
4	18	29	16	31
*p* value	0.098		0.039^*∗*^	
N factor				
0	42	38	46	37
1	28	34	32	30
2	15	32	10	37
*p* value	0.047^*∗*^		<0.0001^*∗∗∗*^	
M factor				
0	73	86	75	84
1	15	18	13	20
*p* value	1.000		0.448	
Stage				
Early	42	33	43	32
Late	46	71	45	72
*p* value	0.027^*∗*^		0.012^*∗*^	

^*∗*^ *p* < 0.05; ^*∗∗∗*^ *p* < 0.001.

**Table 2:** The correlation of IDO1 and TDO2 expression in tumor tissues of CRC patients.

	IDO1		*p*-value
	Low (*n* = 88)	High (*n* = 104)	
TDO2			
Low (*n* = 88)	61	27	
High (*n* = 104)	27	77	<0.0001^*∗∗∗*^

^*∗∗∗*^ *p* < 0.001.

**Table 3:** Association of serum Kyn and Trp levels and clinical parameters of CRC patients.

Parameters	Kyn (nM)	*p* value	Trp (*μ*M)	*p* value
Age				
≦65	2244.16 ± 993.16	0.045^*∗*^	56.84 ± 17.50	0.198
>65	2950.58 ± 2007.58		53.67 ± 18.45	
Gender				
Female	2631.06 ± 2078.19	0.073	54.20 ± 20.27	0.482
Male	2592.51 ± 924.06		56.30 ± 15.07	
Stage				
Early	2574.66 ± 1167.26	0.629	57.62 ± 21.73	0.453
Late	2631.33 ± 1824.91		54.02 ± 15.95	
T factor				
1 and 2	2064.54 ± 724.21	0.202	63.13 ± 15.93	0.052
3 and 4	2714.12 ± 1736.76		53.72 ± 18.03	
N factor				
0	2346.26 ± 974.23	0.444	59.31 ± 19.74	0.063
1 and 2	2767.54 ± 1908.39		52.79 ± 16.57	
M factor				
0	2526.99 ± 1407.94	0.548	55.65 ± 17.91	0.747
1	3080.34 ± 2568.14		52.64 ± 18.78	
IDO1				
−	2756.97 ± 1804.15	0.459	53.77 ± 15.04	0.683
+	2525.70 ± 1534.37		56.04 ± 19.61	
TDO2				
−	2483.46 ± 998.84	0.685	52.88 ± 14.86	0.356
+	2703.55 ± 1966.34		56.79 ± 19.83	
MSI				
Low	2476.90 ± 1773.02	0.037^*∗*^	56.06 ± 18.04	0.291
High	3102.20 ± 1322.23		58.88 ± 15.36	

^*∗*^ *p* < 0.05.

**Table 4:** Correlation of IDO1 and TDO2 expression and gene mutations of CRC patients.

Genes	IDO1		TDO2	
	Low	High	Low	High
*APC*				
Wild type (57.6%)	48	51	48	51
Mutant (42.4%)	30	43	31	42
*p* value	0.356		0.444	
*TP53*				
Wild type (82.0%)	60	81	66	75
Mutant (18.0%)	18	13	13	18
*p* value	0.162		0.693	
*KRAS*				
Wild type (78.5%)	60	75	59	76
Mutant (21.5%)	19	19	21	17
*p* value	0.583		0.269	

**Table 5:** Correlation of IDO1 and TDO2 expression and distribution of beta-catenin protein in CRC patients.

	IDO1		TDO2	
	Low	High	Low	High
Beta-catenin				
Membrane (45.3%)	48	39	47	40
Cytoplasm/nuclear (54.7%)	40	65	41	64
*p* value	0.020^*∗*^		0.043^*∗*^	

^*∗*^ *p* < 0.05.

**Table 6:** Correlation of IDO1, TDO2 expression, and MSI of CRC patients.

MSI	IDO1		TDO2	
	Low	High	Low	High
Low (84.9%)	62	101	64	99
High (15.1%)	16	13	14	15
*p* value	0.102		0.414	
